# The Effect of Once-Daily Gabapentin Extended Release Formulation in Patients With Postamputation Pain

**DOI:** 10.3389/fphar.2019.00504

**Published:** 2019-05-15

**Authors:** Nebojsa Nick Knezevic, Tabish Aijaz, Kenneth D. Candido, Svetlana Kovaleva, Alexei Lissounov, Ivana Knezevic

**Affiliations:** ^1^Department of Anesthesiology, Advocate Illinois Masonic Medical Center, Chicago, IL, United States; ^2^Department of Anesthesiology, The University of Illinois at Chicago, Chicago, IL, United States; ^3^Department of Surgery, The University of Illinois at Chicago, Chicago, IL, United States

**Keywords:** gabapentin, extended release, postamputation pain, chronic pain, amputation

## Abstract

**Objectives:**

To compare gabapentin extended-release, a gastro-retentive formulation, in relieving postamputation pain among gabapentin-experienced and gabapentin-naïve patients.

**Design:**

Open-labeled pilot study.

**Subjects:**

Sixteen patients with postamputation pain (8 patients in the gabapentin-experienced and 8 patients in the gabapentin-naïve groups).

**Methods:**

Patients were started on gabapentin extended-release and were followed up for 8 weeks. Patients reported their pain severity during rest and movement using a numeric rating scale (NRS), interference of pain with daily activities using the modified brief pain inventory (MBPI) questionnaire, and treatment satisfaction using the treatment satisfaction questionnaire for medication (TSQM).

**Results:**

Patients from both gabapentin-experienced and gabapentin-naïve groups achieved a significant and sustainable pain relief over the course of therapy. The pain scores at rest decreased in both gabapentin-experienced and gabapentin-naïve groups from 5.88 ± 1.36 and 4.88 ± 2.95 to 1.88 ± 0.99 and 1.38 ± 1.51, respectively. An average percent of pain relief with gabapentin extended-release was noted to be significant (*p* < 0.01) after 8 weeks of therapy among gabapentin-experienced (81.25 ± 16.42%) and gabapentin-naïve groups (85 ± 17.73%) when compared to baseline for gabapentin-experienced (31.25 ± 29%) and gabapentin-naïve groups (36.25 ± 34.2%), respectively. Gabapentin-experienced and gabapentin-naïve groups had no significant difference in global satisfaction from treatment (79.14 ± 10.47 and 83.3 ± 20.82), convenience of treatment (73.78 ± 19.04 and 90.44 ± 11.66), effectiveness of treatment (72.6 ± 10.1 and 79.73 ± 11.6). The only statistically significant difference among gabapentin-experienced and gabapentin-naïve groups was found in adverse event tolerability (65.78 ± 10.36 and 85.8 ± 10.14, *p* < 0.01).

**Conclusion:**

Once-daily dosing of gabapentin-extended release showed significant improvement in pain severity and functional status, with no difference found between gabapentin-experienced versus gabapentin-naïve patients.

## Introduction

Postamputation pain (PAP) is defined as pain developing after a surgical amputation of a body part, which persists for several months after amputation and for which other causes of pain have been ruled out (Davis, 1993; Crombie et al., 1998). PAP is considered to be primarily a neuropathic type of pain, with its origins within both the central and peripheral nervous systems (Coderre et al., 1993; Nikolajsen et al., 2000; Melzack et al., 2001; Bittar et al., 2005; Flor, 2008; Foell et al., 2014). PAP has emerged as a primary predictor of patient’s health and quality of life following surgery (Doth et al., 2010). The incidence of PAP has been reported to be 33–80% (Richardson et al., 2006; Schley et al., 2008; Wilson et al., 2008; Dworkin et al., 2010b). This wide range is due to the wide variations in the studied populations and varied definitions of PAP (Richardson et al., 2006; Hunter et al., 2008; Schley et al., 2008; Wilson et al., 2008; Dworkin et al., 2010b; Kelle et al., 2017). There is a huge economic impact of PAP, as pain management is the biggest cost factor in the long-term care of these patients (Richardson et al., 2006; Schley et al., 2008; Wilson et al., 2008; Dworkin et al., 2010b). This additional cost is not only due to direct impact on patient’s health and increased demand on healthcare resources, but is also indirectly due to burden on the patient’s family and caregivers (Dworkin et al., 2010a; Duenas et al., 2016). However, the efficacies of current treatment modalities are questionable, given the low satisfaction rates reported widely in the literature (Finnerup et al., 2005; Oster et al., 2005; Rathmell and Kehlet, 2011).

Since PAP is considered to be a neuropathic pain, there is a special interest in using gabapentin for prevention and treatment of pain (Taylor et al., 1998). However, the adoption of gabapentin for the treatment of PAP is slow due to its high rate of adverse events, particularly at the initiation of therapy, which are responsible for a high rate of treatment abandonment in the early stages of implementation (Parsons et al., 2004). The side effect profile of gabapentin has been attributed to its short half-life, resulting in frequent dosing at short intervals and unpredictable absorption from the gut, causing inconsistent levels in the blood and making its titration difficult. A new formulation of gabapentin known as gastro-retentive or extended-release (ER) gabapentin has been developed to provide a slow rate of absorption from the gastrointestinal tract, which would result in increased tolerability of side effects without affecting its efficacy (Depomed, 2007). Gabapentin ER has been extensively studied for the treatment of postherpetic neuralgia. It has shown to be an effective analgesic, with lower rates of adverse events as compared to gabapentin immediate-release (IR) (Irving et al., 2009; Jensen et al., 2009; Sang et al., 2013). To date, no previous study has investigated the use of gabapentin ER for the treatment of PAP. For this purpose, we designed the present study to evaluate the efficacy and safety of gabapentin ER in relieving PAP. To evaluate the safety of gabapentin ER, we compared patients who were new to gabapentin therapy with patients who had taken gabapentin IR.

## Materials and Methods

This study was approved by the Advocate Health Care Institutional Review Board (IRB) and IND exemption was obtained from Food and Drug Administration (FDA). This was an open-labeled, single intervention study determining the efficacy and safety of gabapentin ER (Gralise^®^, Kansas) use in patients experiencing PAP, which was defined as pain after amputation persisting for more than 6 months (Depomed, 2007). Written informed consent was obtained from all patients. Patients were included if they were older than 18 years and suffering from PAP, following limb amputation surgery due to peripheral vascular disease.

Patients were excluded if they met at least one of the following criteria: known allergic reaction to gabapentin, history of epilepsy/seizure disorder, dementia or any cognitive disorder interfering with assessment of pain or adverse reactions. Patients with severe cardiopulmonary disease, uncontrolled hypotension, uncontrolled hypertension, underlying liver disorder, untreated alcohol abuse, chronic diarrhea, dyspepsia, gastroduodenal ulcers, previous gastric reduction surgery, and chronic kidney disease requiring hemodialysis were also excluded from the study. To evaluate the safety of gabapentin, the patient population was divided into two cohort groups based on previous use or lack thereof of gabapentin, i.e., gabapentin-naïve and gabapentin-experienced. Gabapentin-naïve groups included patients with no previous history of gabapentin use, whereas the gabapentin-experienced group included patients who had taken gabapentin IR. Data gathered from the patients included patient characteristics such as age, sex, race/ethnicity, weight, height, body mass index, site of amputation and time duration since amputation in months. In accordance with Initiative on Methods, Measurement, and Pain Assessment in Clinical Trials (IMMPACT) recommendations, we used multiple endpoints in our trial, including an 11-point numeric rating scale (NRS) for pain intensity at rest and during movement which scores pain between 0 and 10 (Turk et al., 2008; Perrot and Lanteri-Minet, 2019). Interference in daily activities due to pain as well as relief provided by pain medications were assessed using the modified brief pain inventory (mBPI) questionnaire. The mBPI measures pain interference by calculating the mean of score from seven activities, i.e., general activity, walking, work, mood, enjoyment of life, relations with others, and sleep. The treatment satisfaction questionnaire for medication (TSQM) was used to assess the patient’s perception of the treatment by measuring its effectiveness, tolerability of adverse events, convenience of therapy, and overall satisfaction from the treatment. mBPI and TSQM have been validated for the use in neuropathic pain syndromes and are routinely used in clinical trials to determine the efficacy of treatment (Zelman et al., 2005; Serpell et al., 2014; Perrot and Lanteri-Minet, 2019).

**FIGURE 1:**
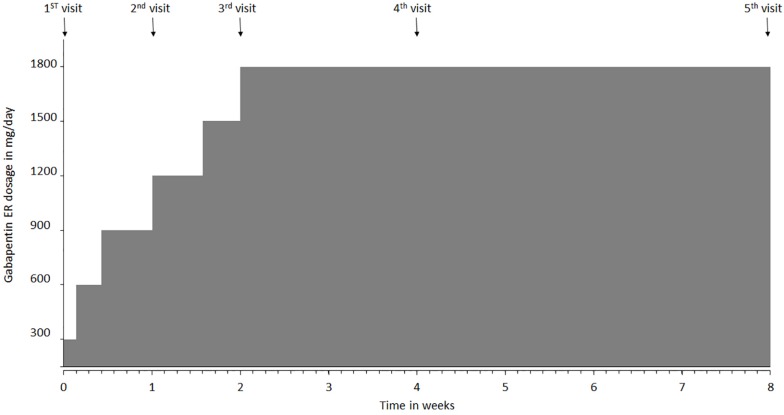
Gabapentin extended-release (ER) titration schedule and clinic visit intervals over the course of the study. The first visit was conducted prior to starting gabapentin ER medication. Patients were instructed to take 300 mg at night, then 600 mg dose on day 2, and 900 mg on day 3 which was continued until day 6. Dosage was increased to 1200 mg on day 7 (visit 2), followed by increase to 1500 mg on day 11, and to 1800 mg (max dose) on day 15, after their visit 3. Visit 4 occurred at week 4, visit 5 at week 8 and visit 6 at week 10.

All the patients participating in the study were started on a once-daily dose of gabapentin ER using a titrating regimen, recommended by the United States Food and Drug Administration (FDA) for the treatment of postherpetic neuralgia (FDA, 2011). Patients who were already on gabapentin underwent a washout period of 2 weeks before starting the trial. The dose was administered with an evening meal in order for the peak dose to occur in the early morning (Depomed, 2007). Patients were instructed to start gabapentin ER at 300 mg/day dosing, gradually increasing the dose up to 1800 mg/day over 2 weeks and continuing the dose of 1800 mg/day for the next 6 weeks to complete the 8-week study period (Figure 1). Patients had a total of 6 visits, with the first visit after washout period and before starting medication. It was followed by visits at week 1, week 2, week 4, and week 8. If patients did not desire to continue using gabapentin ER, the medication was withdrawn after a gradual downward titration over the next 8 days. Gabapentin ER was tapered down to 1200 mg per day for 4 days, followed by 600 mg per day for next 4 days and was stopped after that. We also had a follow-up visit at week 10 to evaluate patients after the completion of the study. This visit was meant to ensure safe withdrawal from gabapentin ER at the end of the study if so desired by the patients (Figure 1). Patients filled an mBPI questionnaire at the beginning and end of the study. During each visit, patients reported pain at rest and pain during movement using the NRS scale, along with any new adverse event that occurred since the previous visit. Patients were also administered the Columbia-Suicide Severity Rating Scale (C-SSRS) at each visit because increased suicidal ideation risk associated with gabapentin (Mula et al., 2013; FDA, 2017; Ghaly et al., 2018). Upon completion of the study, the treatment satisfaction questionnaire for medication was administered. Patients were allowed to continue using gabapentin ER after completion of the study if they were satisfied with the pain relief provided by the drug.

### Statistical Analysis

Descriptive analysis was conducted for the baseline characteristics, i.e., proportion for categorical data and mean with standard deviation for continuous data. The statistical differences in baseline characteristics between the gabapentin-experienced and gabapentin-naïve groups were compared using Pearson’s chi-squared test or Fischer’s exact test for categorical data, and a *t*-test for independent samples for continuous data. The one-way repeated measure analysis of variance (ANOVA) test was used to compare the mean differences in pain at rest and pain during movement with time, with statistical significance set at the 5% level. An independent samples *t*-test was run to determine if there were differences in pain interference and pain relief with medication between gabapentin-experienced and gabapentin-naïve groups. The differences in effectiveness, adverse events, convenience, and overall satisfaction between gabapentin-naïve and gabapentin-experienced groups were compared using a *t*-test for independent samples. Statistical significance was set at the 5% level for all the results. Data analysis was performed using SPSS 23.0 (IBM Corp. Released 2014. IBM SPSS Statistics for Windows, Version 23.0. Armonk, NY: IBM Corp.).

## Results

Eight patients suffering from PAP were recruited to each group of the study according to previous history of gabapentin use. This resulted in 6 males and 2 females being recruited in each group. No statistically significant difference was found in age, BMI, race/ethnicity, amputation site, or for time since amputation between gabapentin-naïve and gabapentin-experienced groups as shown in Table 1. In the gabapentin-experienced group, 3 patients were on gabapentin at the time of enrollment and only 1 patient was on concomitant opioid treatment, while no patient in gabapentin-naïve group was using opioid treatment. Use of non-opioid pain medications such as meloxicam, ibuprofen, acetaminophen, was more common in gabapentin-experienced group with 3 patients on such treatment, while only 1 patient was using non-opioid pain medications in gabapentin-naïve group.

**TABLE 1:** Baseline demographics and characteristics.

**Variable**	**Gabapentin-experienced**	**Gabapentin-naïve**	***p*-value**
**Sex**			1.00
Male	6	6	
Female	2	2	
**Race**			0.411
Caucasian	2	4	
Hispanic	1	0	
African-American	5	4	
**Amputation site**			0.179
Toe unilateral	2	2	
Toe bilateral	1	0	
Below knee	2	5	
Above knee	3	0	
Upper extremity	0	1	
Age (years)	55.5 ± 11.1	55.4 ± 9.8	0.981
Body mass index kg/m2)	30.8 ± 5.9	29 ± 2.4	0.441
Time since amputation (months)	133.2 ± 142.7	100.6 ± 79.8	0.581

**FIGURE 2:**
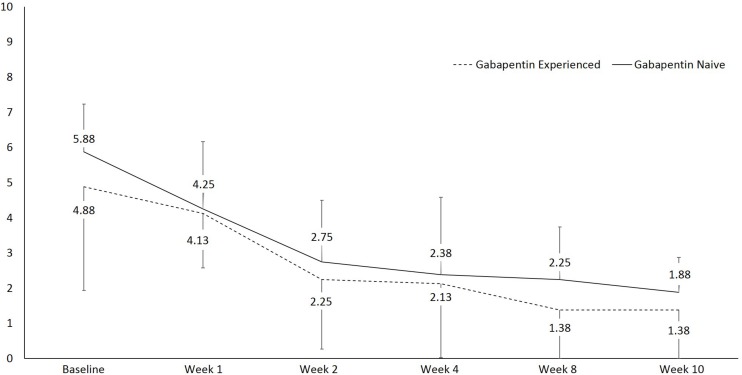
Line graph showing pain at rest using numeric rating scale (NRS) over the course of the study.

**FIGURE 3:**
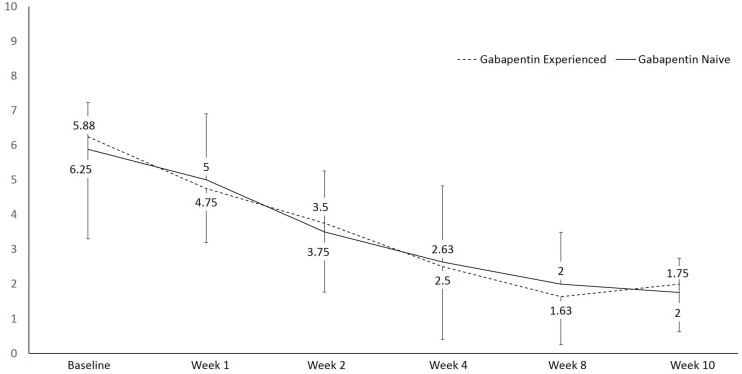
Line graph showing pain during movement using numeric rating scale (NRS) over the course of the study.

There was a statistically significant difference in pain at rest during the different visits, *F*(1, 15) = 27.6, *p* < 0.001, partial η^2^ = 0.648 (Figure 2). Within group analysis, using repeated measures with Bonferroni adjustment, significant difference in pain at rest was shown on 3rd visit compared to 1st and 2nd visits, which persisted until the 6th visit. Similarly, a statistically significant difference in pain during movement was seen during the different visits, *F*(1, 15) = 49.7, *p* < 0.001, partial η^2^ = 0.768 (Figure 3). Within group analysis, using repeated measures with Bonferroni adjustment, significant differences in pain during movement were identified on the 3rd visit compared to 1st and 2nd visit, which persisted until the 6th visit.

**FIGURE 4:**
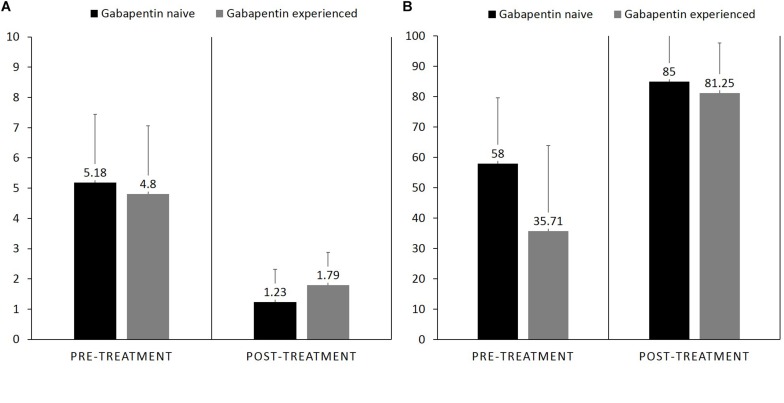
Bar graph showing difference in pre-treatment and post treatment, **(A)** pain interference between gabapentin naïve vs. gabapentin experienced patients who completed the study, **(B)** pain relief with medication.

Pain interference reported by gabapentin-experienced patients was (*M* = 4.8, *SD* = 2.2) and gabapentin-naïve patients at the end of study was (*M* = 5.1, *SD* = 2.2), with no statistically significant difference between the groups, *MD* = −0.55, 95% CI [−2.43, 1.33], *p* = 0.54. Pain relief with medications reported by gabapentin-experienced patients at the end of study was (*M* = 35.71, *SD* = 28.2) and gabapentin-naïve patients was (*M* = 58.0, *SD* = 21.7), with no statistically significant difference between the groups, *MD* = 8.5, 95% CI [−14.5, 22.1], *p* = 0.67 (Figure 4).

**FIGURE 5:**
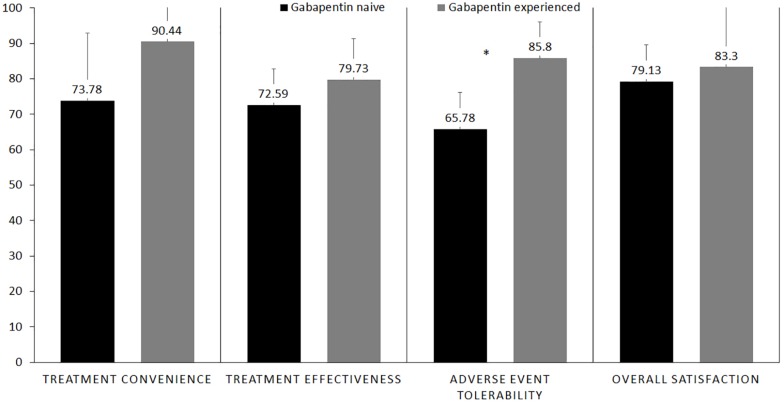
Bar graph showingfour aspects of treatment satisfaction questionnaire for medication (TSQM) in gabapentin-naïve vs. gabapentin-experienced patients who completed the study. *Statistical significant.

The gabapentin-experienced group reported convenience of therapy at 90.4% and effectiveness at 79.7% compared to 73.8% for convenience of therapy and 72.6% for effectiveness reported by the gabapentin-naïve group. There were no significant differences in the proportions of convenience (*p* = 0.53) and effectiveness (*p* = 0.21) between the two groups. Gabapentin-experienced group reported acceptable tolerance of adverse effect at 85.8% as compared to 65.8% in the gabapentin-naïve group, which was a statistically significant difference, *p* = 0.002. Gabapentin-naïve group scored 83.3% in the overall satisfaction associated with the therapy, compared to 79.1% score in the gabapentin-experienced group, however, there were no significant differences between the two groups, *p* = 0.62 (Figure 5). Adverse events reported by the patients at each visit are mentioned in Table 2.

**TABLE 2:** Adverse events reported at each visit by the patients.

	**Week 1**	**Week 2**	**Week 4**	**Week 8**	**Week 10**
Dizziness	3	3	4	1	0
Headache	1	0	0	1	0
Diarrhea	1	2	1	3	1
Somnolence	1	2	2	2	1
Vivid dreaming	0	1	0	0	0
Occasionalreflux	0	1	0	0	0
Nausea	0	1	1	2	0
Dry mouth	0	0	0	1	0
Blurry vision	0	0	0	1	0

## Discussion

Our study found a significant reduction in PAP and interference of pain with daily functions after initiation of gabapentin ER therapy in both gabapentin-experienced and gabapentin-naïve patients. Our results are in line with other studies on the use of gabapentin ER in neuropathic pain showing a similar reduction in pain severity (Irving et al., 2009; Beal et al., 2012). Gabapentin ER has consistently shown improvement in postherpetic neuralgia pain in several clinical trials, leading to its approval by the FDA in 2011 (FDA, 2011). Since its approval, phase 3 clinical trial testing has proven it to be safe and effective at a dose of 1800 mg/day for the treatment of postherpetic neuralgia (Irving et al., 2009; Jensen et al., 2009; Beal et al., 2012; Rauck et al., 2013; Sang et al., 2013; Kaye et al., 2014). To date, very few studies have evaluated the role of gabapentin ER in the treatment of other chronic neuropathic pain conditions (Sandercock et al., 2012; Kaye et al., 2014; North et al., 2016). There is limited data supporting the use of gabapentin ER to treat pain due to spinal stenosis, fibromyalgia and diabetic neuropathy (Sandercock et al., 2012; Kaye et al., 2014; North et al., 2016). So far, no research has been carried out to evaluate the use of gabapentin ER for the treatment of PAP.

To better understand the role of gabapentin in the treatment of PAP, it is important to understand its molecular structure and mechanism of action. Gabapentin, [1-(aminomethyl) cyclohexane acetic acid], is a synthetic analog of gamma-aminobutyric acid (GABA), which subsequently inhibits pain neurotransmission, particularly targeting neuropathic pain pathways (Taylor et al., 1998). Several hypotheses have been proposed to explain the mechanism of action of gabapentin, one of which is modulation of sodium or calcium channels, but there is limited evidence to support this hypothesis (Brown et al., 1996; Moore et al., 2002; Chen and Mao, 2013). It is known that gabapentin has effects on the central nervous system via increased serotonin concentrations, which is partly responsible for its pharmacodynamic effect. Another explanation is the inhibition of the ventrolateral periaqueductal gray pathway in amygdala, which is considered crucial for its anticonvulsant effects and possibly contributive to its role in the alleviation of pain and anxiety (Tupal and Faingold, 2012). Some investigators have proposed a different mechanism, which involves the noradrenergic pathway by binding to the alpha-2 delta subunit in neural tissues to inhibit dorsal root ganglion transmission (Alden and Garcia, 2001).

Gabapentin ER has been evaluated as both once-daily and twice daily dosing in the past. In our study, we used a once-daily dosage regimen only because previously published literature has not shown any differences in efficacy with either dosing regimen (Wang and Zhu, 2017). This has been confirmed by a meta-analysis conducted to compare different dosing regimens of gabapentin in the treatment of postherpetic neuralgia, which found that the once-daily dosing regimen of gabapentin ER is as effective as the twice daily dosing regimen. Another study by North et al. (2016) evaluated the safety and titration regimen of gabapentin ER in patients with fibromyalgia. Similar to our study, they also compared gabapentin-naïve vs. gabapentin-experienced patients and found encouraging results. Titration regimen, upper dose limit and safety profile was comparable to our study results (North et al., 2016). In a study of pharmacokinetics by Chen et al. (2011) gabapentin ER exhibited superior dose linearity compared with the gabapentin IR dosage form that allows for a reduction in dosing frequency, allowing a once-daily regimen.

Our results on gabapentin ER adverse effect tolerance were very encouraging. Most common adverse events in our study were dizziness, somnolence, and headache, which are consistent with the previously published literature (Beal et al., 2012). Rates of adverse events in our study were similar to previously published literature, which proves that gabapentin ER has good tolerability in patients suffering from PAP. Pharmacokinetic properties such as slower increase to a flatter peak may diminish the occurrence of adverse events compared with gabapentin IR. It also has a slower increase to the peak concentration and less drastic fluctuations in blood concentrations, which might possibly reduce the rate of adverse events compared to the IR formulation (Gordi et al., 2008; Kagan and Hoffman, 2009; Chen et al., 2011, 2013). The polymers in the gabapentin ER tablet are commonly used in the food industry and have not been reported to be associated with toxicological issues (Berner and Cowles, 2006). The intrasubject variability in gabapentin absorption is substantially less than that of the intersubject variability; thus gabapentin plasma level is dependent on dose (Gidal et al., 2000).

We showed that gabapentin is safe to use as up to a dose of 1800 mg/day in patients with PAP. We also demonstrated that it is safe to titrate gabapentin to its maintenance dose over a 2-week period in both gabapentin-naïve and gabapentin-experienced patients. For this reason, the FDA recommended dose titrations up to 1800 mg/day, over 2 weeks, at the time of approval of gabapentin ER for postherpetic neuralgia (FDA, 2011). Once-daily dosing of 1800 mg/day is a standard dose of gabapentin ER used for postherpetic neuralgia (Fan et al., 2014). Safety profile of gabapentin ER was studied in patients suffering from postherpetic neuralgia, where it was found to be safe with good adverse effect tolerability at doses of up to 1800 mg/day which were used in our study (Irving et al., 2009; Jensen et al., 2009). Gabapentin ER has an extended duration and results in less fluctuations in blood concentrations, making it possible to use lower daily doses as compared to gabapentin IR (Kagan and Hoffman, 2009). It is important to remember that gabapentin IR has been used at doses of up to 3600 mg/day with no significant changes in the adverse event profile (Backonja and Glanzman, 2003). To date, no study has been published comparing gabapentin ER and IR formulation at doses higher than 3000 mg/day. Cowles et al. (2012) have used gabapentin ER in postmenopausal women with hot flashes with a daily dose of 3000 mg with no significant differences in the adverse effect profile. This study was initiated in February 2005 following positive results from a phase 1 trial in which gabapentin ER demonstrated a pharmacokinetic profile suitable for twice-daily dosing. In two pharmacokinetic studies, gabapentin ER achieved improved bioavailability at higher doses (Depomed, 2007).

Our study also demonstrated that gabapentin ER is effective in relieving PAP. Our study was designed to compare the role of gabapentin ER between gabapentin-naïve and gabapentin-experienced patients; however, we neither had a placebo nor other medication group, which could have served as true control group(s). There is limited literature available comparing gabapentin with placebo in PAP. A noteworthy trial was conducted by Smith et al. comparing gabapentin with placebo in patients with PAP, which did fail to show significant pain relief in patients on gabapentin (Smith et al., 2005). However, the baseline pain scores were significantly lower as compared to our populations, which might explain this difference. Bone et al. (2002) showed a significant improvement in pain scores with the use of gabapentin in patients with postamputation phantom pain only. In this study, baseline pain intensity and percentage of pain relief with gabapentin were similar to our trial. We believe that the results from these two studies support our inference that gabapentin ER is similar in efficacy as compared to gabapentin IR. Recently, another formulation of gabapentin, gabapentin enacarbil, has been approved as once-daily dose for the treatment of postherpetic neuralgia (Backonja et al., 2011; Harden et al., 2013). Similar to Gabapentin ER, Gabapentin enacarbil has been shown to be safe and effective up to doses of 2400–3600 mg/day in patients suffering from postherpetic neuralgia (Calkins et al., 2016). We believe that both gabapentin ER and gabapentin enacarbil have the potential to replace gabapentin IR; however, a direct comparison between the two formerly described extended duration formulations is needed before reaching any conclusions.

Our study limited the maximum dose of gabapentin ER to 1800 mg/day, which is equivalent to the commonly used dosage of gabapentin IR for the treatment of PAP. However, it is important to determine the maximum dose, which can be used to treat PAP without increasing risks of serious adverse effects. Secondly, we excluded patients with end-stage renal disease because of the known renal metabolism and excretion of gabapentin, which would require a dosage adjustment and because of a potential factor for bias in interpreting the attained results. Although gabapentin ER has been shown to be safe in patients with end-stage renal disease in a previous randomized control trial, further confirmation of the safety of gabapentin ER in this subgroup is needed. Lastly, it is also important to consider doing a long-term follow-up study to assess whether the effects of this medication in this patient population is sustainable over long period of time. Furthermore, costs of gabapentin ER therapy should be considered before deciding its role in the treatment of PAP. We therefore suggest that a detailed analysis of total costs associated with gabapentin therapy be evaluated, which is possibly only done in a large clinical trial aimed at assessing patients over a longer period of time than what has been accomplished to date.

## Conclusion

We found that a gabapentin ER formulation both alleviates the pain severity and improves the functional status of patients suffering from PAP. Moreover, we did not find any new or unusual adverse events from the use of gabapentin ER. We are extremely optimistic that gabapentin ER may be of great value clinically for patients suffering from PAP.

## Ethics Statement

Study was approved by Advocate Healthcare Institutional Review Board.

## Author Contributions

NK and KC designed the study, revised the manuscript, and made the final corrections. NK, AL, and IK collected the data. AL, TA, and IK analyzed the data and interpreted the results. SK and TA wrote the manuscript. All authors approved the final version of the manuscript.

## Conflict of Interest Statement

Depomed, Inc. provided Gralise medication for the study purpose without any charge to the subjects/subjects’ insurance.

The authors declare that the research was conducted in the absence of any commercial or financial relationships that could be construed as a potential conflict of interest.
